# Impact of time-related factors on biologically accurate radiotherapy treatment planning

**DOI:** 10.1186/s13014-018-0973-6

**Published:** 2018-02-23

**Authors:** Yushi Wakisaka, Masashi Yagi, Iori Sumida, Masaaki Takashina, Kazuhiko Ogawa, Masahiko Koizumi

**Affiliations:** 1 Department of Medical Physics & Engineering, Osaka University Graduate School of Medicine, Suita, Osaka Japan; 2 Department of Carbon Ion Radiotherapy, Osaka University Graduate School of Medicine, Suita, Osaka Japan; 3 Department of Radiation Oncology, Osaka University Graduate School of Medicine, Suita, Osaka Japan

## Abstract

**Background:**

The incomplete repair (IR) model expresses the cell repair effect from radiation-induced damage over time, which is given little consideration in actual treatment planning. By incorporating the IR model into the normal tissue complication probability (NTCP), the accuracy and safety of treatment plan evaluations concerning the effect of repair can be improved. This study aims to evaluate the impact of incorporating the IR model into the NTCP by varying time-related factors such as the repair half-time (T_1/2_) and the junction-shift sc3hedule in craniospinal irradiation (CSI).

**Methods:**

CSI was planned retrospectively, and the NTCP of the spinal cord was calculated with the IR model for values of T_1/2_ from 1 to 10 h. The NTCP in the case of changing the junction-shift schedule was also examined in the same manner.

**Results:**

The NTCP with the IR model increased with increasing T_1/2_, which is prominent for the larger T_1/2_. By changing the junction-shift schedule, the NTCP with the IR model decreased when adjacent fields overlapped.

**Conclusions:**

The IR model is a valuable addition to treatment planning because it enables the NTCP to be evaluated including the effect of repair and differences in scheduling to be reflected in the NTCP. However, these are largely dependent on the value of the T_1/2_.

## Background

Currently, physical dosimetric information such as the dose–volume histogram and dose distribution are used to evaluate treatment planning. In addition, radiobiological treatment planning has great potential to estimate the treatment outcome [1]. Although the normal tissue complication probability (NTCP) is a radiobiological index that can mathematically express the probability of complications, it overlooks several biological effects such as cell repair.

To account for the cell repair effect, Oliver et al. [2] proposed the time-based incomplete repair (IR) model, which was later modified by Levin-Plotnik et al. [3], to include successive doses. By incorporating this model into the NTCP formula, the probability of complications, including the cell repair effect, can be estimated. Many authors have studied the cell repair effect, particularly in the spinal cord. Several studies have considered the repair half-time, an important parameter of the IR model, by investigating the kinetics of repair in a rat’s spinal cord [4–6]. However, the reported values of repair half-time are wide ranging and contain uncertainties. For example, a repair half-time of 5.0 h with a wide confidence interval (CI) of 0.6–9.3 h has been reported [4]. Moreover, these data were for rats, not humans. In the present study, we focus on the fact that the repair half-time of the spinal cord is comparatively long and contains uncertainties. We also examine the difference in the NTCP of the spinal cord with the IR model for varying values of repair half-time.

A form of radiotherapy that can potentially induce complications with the spinal cord is craniospinal irradiation (CSI), an essential component in the curative treatment of patients with primitive neuroectodermal tumors such as medulloblastoma and other brain tumors that have an increased risk of leptomeningeal spread [7, 8]. Owing to the planning target volume length in the craniocaudal direction, several fields are generally used to cover the whole target volume. The junction-shift technique is used to smooth any over- or underdose arising at a junction between different fields, which results from small errors in patient set up. However, the junction-shift schedule varies among facilities (e.g., change junction every week, two weeks, or one third of treatment.) [9], and is not covered by any recommendations. Considering that the variations in this schedule have no influence on the dose distribution, the conventional NTCP is calculated using a fixed physical dose distribution. However, we adopt the time-sensitive IR model in the expectation that the NTCP can depend on the junction-shift schedule.

In this study, we performed a planning study to examine the impact on NTCP of the factors related to cell repair, such as the repair half-time and the junction-shift schedule, by applying the IR model to CSI. In addition to a fixed-dose CSI plan, we calculate the NTCP dependence on both repair half-time and dose under the assumption of simple dose distributions.

## Methods

### NTCP with IR model

The cell survival fraction *S* is given by the linear–quadratic (LQ) model as

1 $$ \mathrm{lnS}={\sum}_{k\kern0.5em =\kern0.5em 1}^n\left\{-\alpha {d}_k-\beta {d_k}^2\right\}, $$

where *α* and *β* are the LQ model parameters and *d*_*k*_ is the dose of fraction *k*. Therefore, the Poisson LQ NTCP model based on the relative seriality model [10] and the critical volume model [11] is expressed as follows:

2 $$ {\displaystyle \begin{array}{l} NTCP={\left(1-\prod \limits_{i=1}^M{\left(1-{\left[\mathit{\exp}\left(-{N}_0S\right)\right]}^s\right)}^{v_i}\right)}^{\frac{1}{s}}\\ {}={\left(1-{\prod}_{i\kern0.5em =\kern0.5em 1}^M{\left(1-{\left[\mathit{\exp}\left(-{N}_0\mathit{\exp}{\sum}_{k\kern0.5em =\kern0.5em 1}^n\left\{-\alpha {d}_{k,i}-\beta {d_{k.i}}^2\right\}\right)\right]}^s\right)}^{v_i}\right)}^{\frac{1}{s}},\end{array}} $$

where *M* denotes the total number of voxels, *d*_*k,i*_ is the dose of fraction *k* to voxel *i*, and *n* is the total number of fractions. *N*_*0*_ denotes the initial number of cells, *v*_*i*_ is the relative volume of voxel *i*, and *s* represents the seriality.

To take radiation-induced cell damage into account, we use the IR model [2], which introduces the concept of “dose equivalent of IR.” The idea is that after a dose of size *d*, the injury induced by some fraction *θ* of the dose is still unrepaired by the time the next dose is given. The fraction *θ* is assumed to decay exponentially with time according to

3 $$ \theta =\mathit{\exp}\left(-\mu \Delta t\right), $$

where Δ*t* is the inter-fractional interval and *μ* is the repair constant. Using the repair half-time T_1/2_ as the time needed for half the damaged calls to be repaired, *μ* is expressed as

4 $$ \mu =\frac{\ln 2}{T_{1/2}}. $$

By incorporating the cell repair effect on successive doses [3], the survival fraction derived from the LQ model is given by

5 $$ \ln {S}_{repair}=\sum \limits_{k=1}^n\left\{-\alpha {d}_k-\beta {d_k}^2-2\beta {d}_k\sum \limits_{p=1}^{k-1}{d}_p\prod \limits_{p=q}^{k-1}{\theta}_q\right\}, $$

where *θ*_*q*_ =  *exp* (−*μ∆t*_*q*_), and *Δt*_*q*_ is the time between fractions *q* and *q + 1*. Thereafter, the NTCP including cell repair is expressed as

6 $$ {\displaystyle \begin{array}{l}{NTCP}_{repair}\\ {}={\left(1-\prod \limits_{i=1}^M{\left(1-{\left[\mathit{\exp}\left(-{N}_0\mathit{\exp}\sum \limits_{k=1}^n\left\{-\alpha {d}_{k,i}-\beta {d_{k,i}}^2-2\beta {d}_{k,i}\sum \limits_{p=1}^{k-1}{d}_{p,i}\prod \limits_{p=q}^{k-1}{\theta}_q\right\}\right)\right]}^s\right)}^{v_i}\right)}^{\frac{1}{s}}.\end{array}} $$

NTCP parameters for the spinal cord were reported as *D*_*50*_ = 68.6 Gy, *γ* = 1.90, *α/β* = 3.00, and a seriality of 4.00 [12], which were based on radiation tolerance data for clinical myelitis necrosis [13]. However, this NTCP model should have repair term T_1/2_ potentially because this was derived from clinical fractionated irradiation. On the other study, T_1/2_ was reported to be 5 h with 95% CI of 0.6 – 9.3 h, which is obtained by irradiation to rat spinal cord with variable intervals [4]. In this study, therefore, we examined the dependence of NTCP on T_1/2_ varying from 0 h to 10 h to cover 95% CI on the assumption that NTCP reported in Ref. [12] was given by potentially including T_1/2_ = 5 h, as following procedure.

1. Original NTCP was calculated by using parameters reported in Ref. [12], which is corresponding to NTCP calculated by substituting 0 h for T_1/2_ in eq. (6).
2. Apparent NTCP including repair *NTCP*_*repair*_*(T*_*1/2*_*)* was calculated by eq. (6) for T_1/2_ up to 10 h with 1 h intervals.
3. True NTCP including repair *NTCP*_*repair*_*’(T*_*1/2*_*)* was obtained by shifting *NTCP*_*repair*_*(T*_*1/2*_*)* to *NTCP*_*repair*_*(5 h)* be equal to original NTCP (*NTCP*_*repair*_*(0 h)*) by following equation;

7 $$ {NTCP_{repair}}^{\hbox{'}}\left({T}_{1/2}\right)={NTCP}_{repair}\left({T}_{1/2}\right)-\left[{NTCP}_{repair}(5h)-{NTCP}_{repair}(0h)\right]. $$

**Fig. 1:**
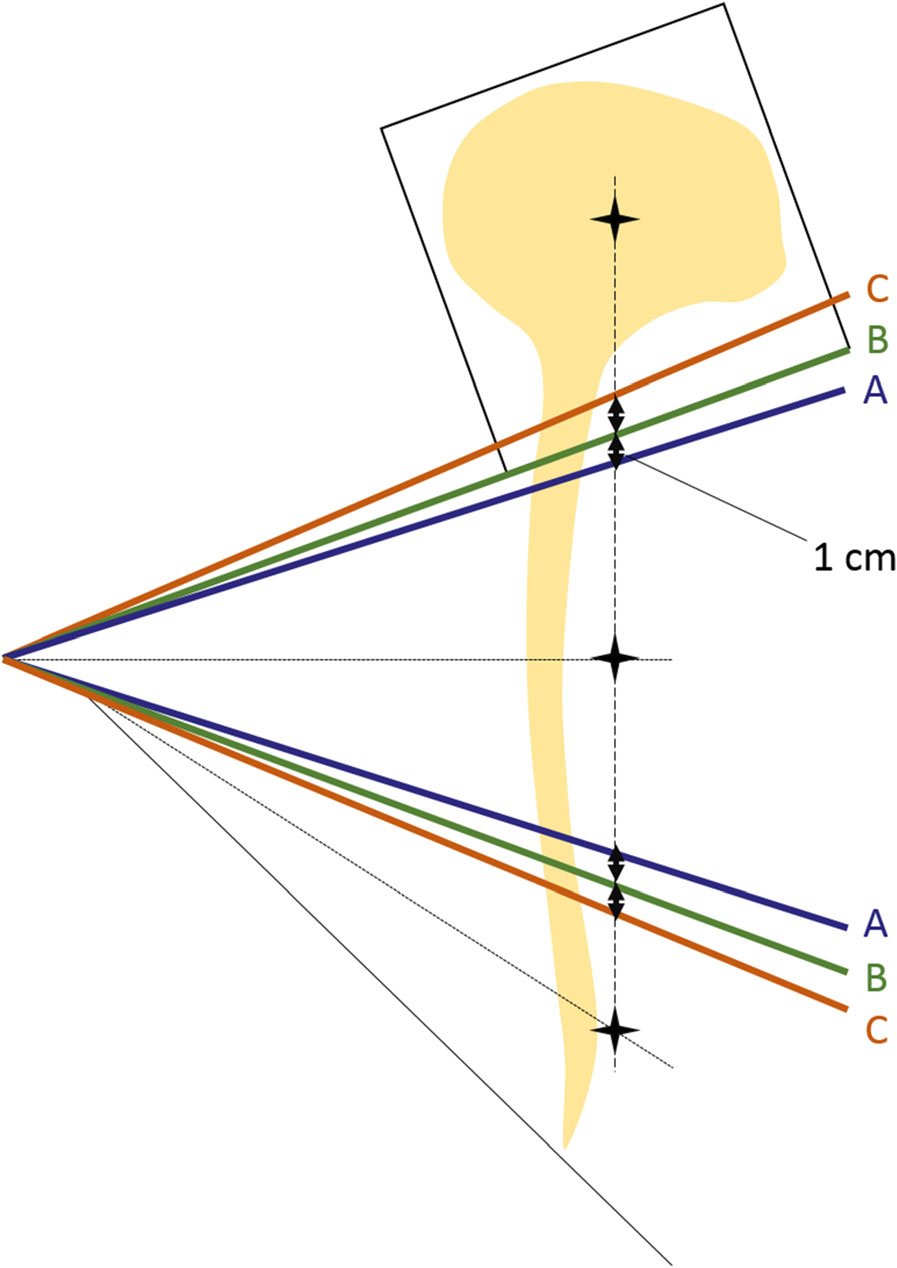
NTCP calculation method on the assumption that NTCP reported in Ref. [12] was given by potentially including T_1/2_ = 5 h, which has no repair term originally

This procedure is also described in Fig. 1.

### Beam arrangement in CSI

RayStation (ver. 7.2.5, RaySearch Laboratories, Stockholm, Sweden) was used to create retrospective 3D conformal radiation therapy plans for six adult patients receiving CSI from a Siemens Artiste linear accelerator with a 6-MV photon beam, in which the dose distribution is calculated by the collapsed cone convolution superposition method [14].

For each patient, we prescribed a total dose of 36 Gy (20 fractions of 1.8 Gy each) to the target with the margin of 5 mm to both the spinal cord and the brain, in which the minimum dose was constrained not to be less than 95% of the prescribed dose. The treatment was assumed to be performed in five fractions every week without weekends, as is usual in clinical practice.

**Fig. 2:**
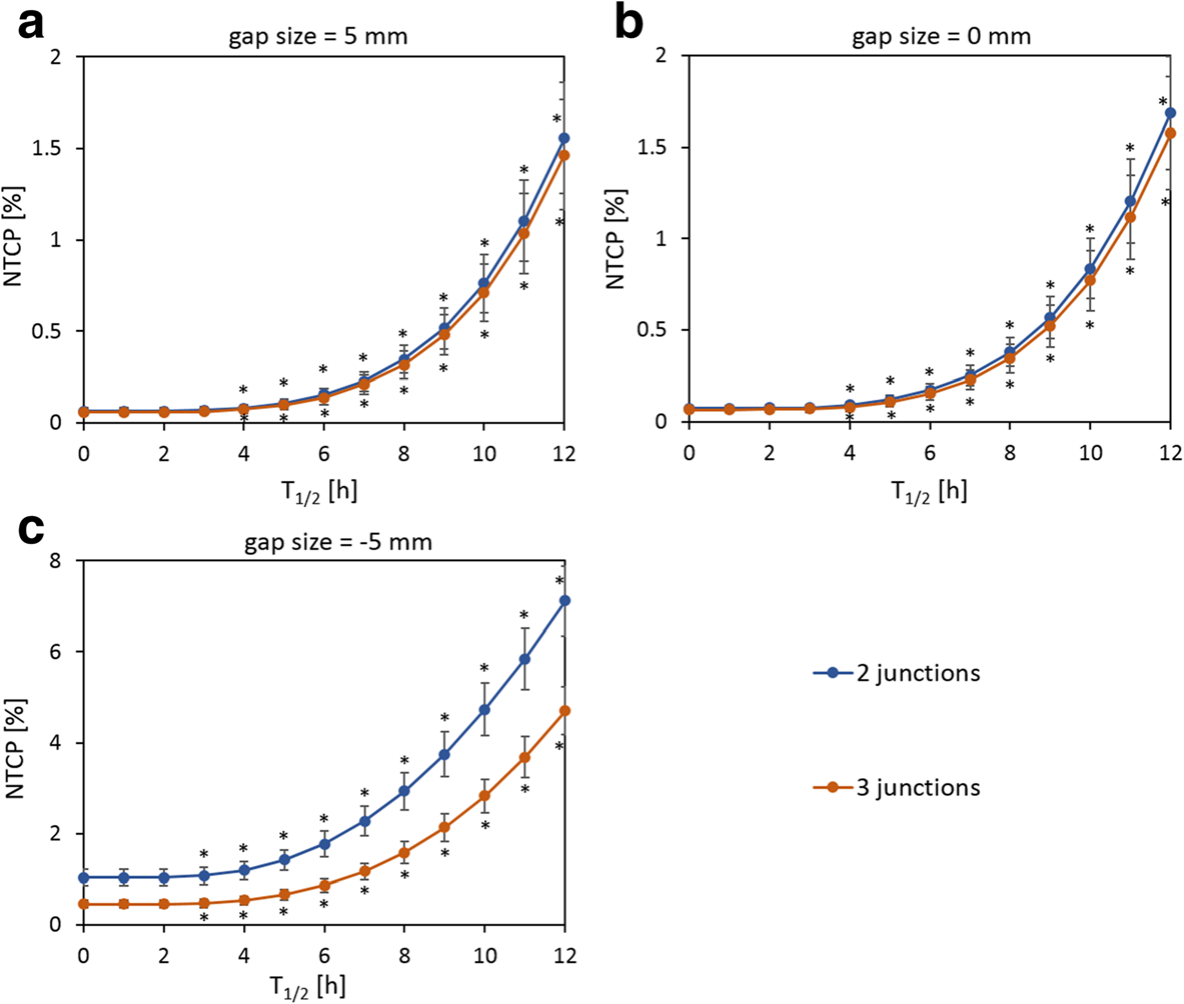
Schematic of the junction-shift technique with no gap. Once junction position B is mathematically determined, positions A and C are achieved by closing or opening two MLC leaves (1 cm) along each isocenter axis

We used two opposing lateral fields to treat the entire brain and two abutted spinal fields to treat the upper and lower spinal fields [15]. The brain-field isocenter was located at the volumetric center of the brain, and we used only a longitudinal shift for the spinal-field isocenters. We used the couch angle to match the brain field with the inferior edges of the two lateral beams, and we rotated the collimator to match the upper spinal-field divergence. For the lower spinal field, we rotated the gantry to match the inferior edge divergence of the upper spinal field with a couch rotation of 90°. To facilitate several types of junction shift, we defined three different junction positions in the case of no gap (i.e., a gap size of zero) (Fig. 2). Junction position B was established to match each field exactly. Junction position A was achieved by closing two multi-leaf collimator (MLC) leaves (1 cm) of each anterior and posterior edge of the upper spinal field and by opening two MLC leaves of each posterior edge of the brain field and the anterior edge of the lower spinal field. Junction position C was achieved by moving the MLC leaves in the opposite directions of those for junction position B.

In addition to the case of no gap, gaps of 5 and − 5 mm (i.e., a 5-mm overlap) were created in the same manner. Although these are worst case scenario of systematic errors, it could arise because of setup errors or patient movement. For example, a mistake in counting a patient’s vertebrae could lead to the treatment area being located erroneously, thus resulting in overlapping junction areas [16].

### Schedules of junction shifts

Using the junction positions defined above, some junction-shift schedules were determined for each gap size assuming five fractions per week (Table 1); this process was based on Ref. [9]. Assuming two junction positions (positions A and B), biweekly, weekly, and daily plans refer to changing the junction every two weeks, every week, and every day, respectively. Assuming three junction positions (positions A, B, and C), a 1/3 treatment plan refers to changing the junction every 1/3 of the treatment (i.e., after 7 and 14 fractions). The simplest schedules among each number of junction positions (i.e., the biweekly plan for two junctions and the 1/3 treatment plan for three junctions) were taken as the reference schedules.

**Table 1:** Schedules of junction shifts considered in this study, with junction positions A, B, and C corresponding to Fig. 2

		Fractions																			
		1	2	3	4	5	6	7	8	9	10	11	12	13	14	15	16	17	18	19	20
The number of junction positions	Schedules of junction shift	Junctions position																			
2	Biweekly plan*	A										B									
	Weekly plan	A					B					A					B				
	Daily plan	A	B	A	B	A	B	A	B	A	B	A	B	A	B	A	B	A	B	A	B
3	1/3 treatment plan*	A							B							C					
	Daily plan	A	B	C	A	B	C	A	B	C	A	B	C	A	B	C	A	B	C	A	B

*reference schedule

### Calculation of NTCP in RayStation

First, to estimate the effect of the value of T_1/2_, the NTCP was calculated with the IR model via the biological evaluation tool RayBiology in RayStation, in which the junction-shift schedule was fixed to the reference schedules for each number of the junction positions.

Second, the NTCP in the case of changing the junction-shift schedule was examined to investigate whether it was affected. For each junction position, the variation in NTCP when changing from the reference schedules to the other schedules was calculated.

### Manual calculation of NTCP

To examine the NTCP dependence on both T_1/2_ and the dose, we calculated the NTCP manually for the spinal cord by assuming many voxels. We began by providing a uniform dose to the entire spinal cord. Thereafter, we gave a uniform dose to 1% of the volume of the spinal cord and no dose to the remaining 99%. To eliminate variations in dose per fraction under a fixed total dose, the total dose was expressed by the equivalent dose in 2 Gy fractions (EQD_2_) and was varied from 30 to 120 Gy. The fractionation schedule (20 fractions/4 weeks) was the same as those for the CSI planning. Given that we assumed each voxel to be exposed to a fixed dose, no variation in junction-shift schedule was considered in this calculation.

### Statistical analysis

We evaluated the statistical significance of the proposed changes in NTCP. Given the small sample size (*n* = 6), we used the Shapiro–Wilk test to validate the NTCP normality. Thereafter, we used a paired Student *t*-test to investigate the statistical significance with a confidence level of 95%.

## Results

### T_1/2_ dependence of NTCP considering IR model

**Fig. 3:**
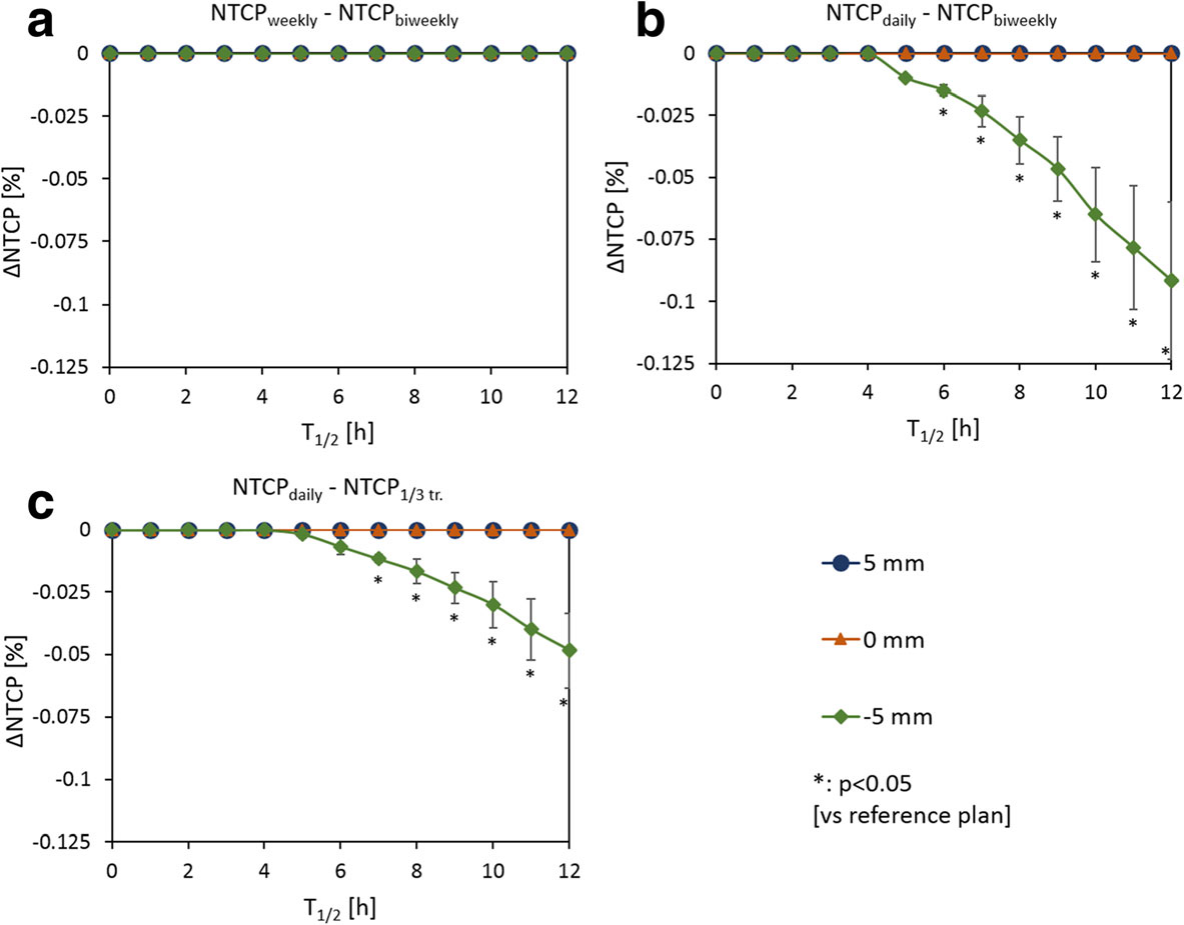
NTCP as a function of T_1/2_ on the reference schedules for two junction positions (blue) and three junction positions (orange) for each gap size: (**a**) 5, (**b**) 0, and (**c**) − 5 mm

Figure 3 shows the NTCP as a function of T_1/2_ on the reference junction-shift schedules (i.e., the biweekly plan for two junction positions and the 1/3 treatment plan for three junction positions). The NTCP was largest with a gap size of − 5 mm, followed by 0 and 5 mm, although the difference between the latter two was small. With the same gap size, three junction positions resulted in a lower NTCP than two junction positions, particularly for a gap size of − 5 mm.

For all gap sizes, the NTCP with the IR model varied depending on T_1/2_. In comparison to the NTCP with T_1/2_ = 5 h, that with the shorter T_1/2_ was significantly smaller, and that with the longer T_1/2_ was significantly larger. In addition, the longer T_1/2_ gave more prominent increase of NTCP, which reached a maximum with T_1/2_ = 10 h.

### Dependence of NTCP on junction-shift schedule with the IR model

**Fig. 4:**
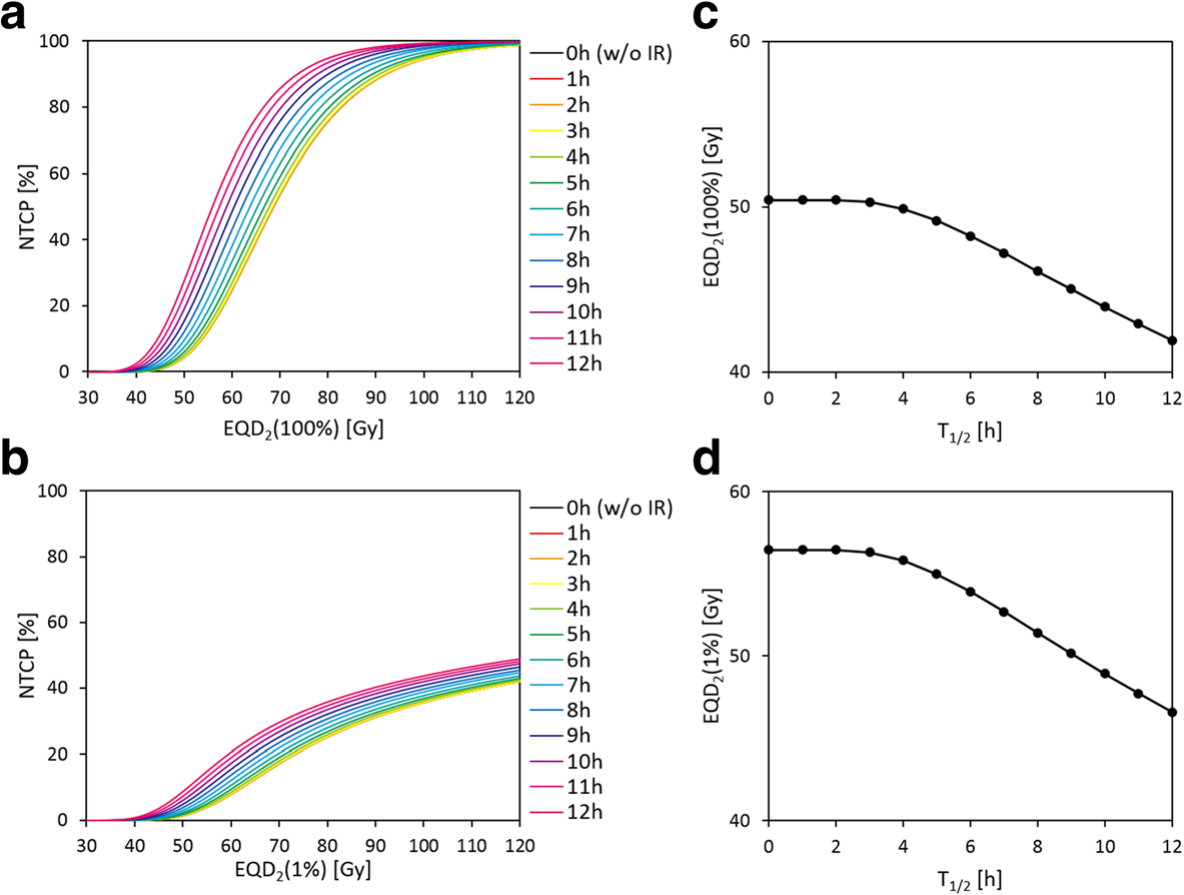
Deviation of NTCP upon changing the junction-shift schedule from a reference schedule to a different schedule: from biweekly to (**a**) weekly and (**b**) daily for two junction positions; (**c**) from 1/3 treatment to daily plan for three junctions. In each case, the blue circles, orange triangles, and green rhombi represent gap sizes of 5, 0, and − 5 mm, respectively

Figure 4 shows the NTCP deviation when the junction-shift schedule was changed from the reference schedules to the other schedules for each number of junctions. For two junction positions, no NTCP reduction was observed upon changing to the weekly plan. However, for a gap size of − 5 mm, a statistically significant deviation of NTCP was seen for T_1/2_ longer than 6 h upon changing to the daily plan; the NTCP deviation increased negatively as T_1/2_ decreased and reached a maximum of − 0.07 (T_1/2_ = 10 h). For three junction positions, the NTCP deviation was statistically significant for T_1/2_ longer than 7 h upon changing to the daily plan for a gap size of − 5 mm; the NTCP deviation increased negatively as T_1/2_ decreased and reached a maximum of − 0.03 (T_1/2_ = 10 h).

### Manual calculation of NTCP with varying T_1/2_ and EQD_2_

**Fig. 5:**
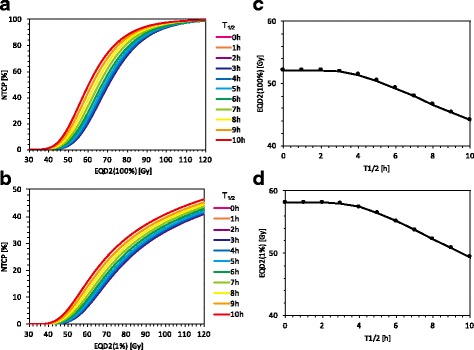
NTCP as a function of EQD_2_ ((**a**), (**b**)), and EQD_2_ that gives an NTCP of 5% as a function of T_1/2_ ((**c**), (**d**)), assuming EQD_2_ was given to 100% ((**a**), (**c**)) and 1% ((**b**), (**d**)) of the volume of the spinal cord

We manually calculated the NTCP curves with the IR model by varying EQD_2_ which is given to 100% or 1% of the volume of spinal cord (Fig. 5). When EQD_2_ was given to the entire spinal cord (Fig. 5(a)), an EQD_2_ of 68.6 Gy gave an NTCP of 50% for T_1/2_ = 0 (i.e., without the IR model). However, the NTCP curve shifted upward upon incorporating the IR model for T_1/2_ longer than ~ 3 h. Moreover, a larger T_1/2_ gave a higher NTCP curve. When EQD_2_ was given to 1% of the volume of the spinal cord (Fig. 5(b)), the NTCP was not saturated with high EQD_2_ because of partial exposure and continued to increase with increasing EQD_2_. Although the NTCP was lower than that when the entire spinal cord was irradiated, it increased as T_1/2_ increased over ~ 3 h, similar to that when the entire spinal cord was irradiated.

Figure 5 also shows the EQD_2_ that gave an NTCP of 5% for various T_1/2_, assuming that EQD_2_ was given to (c) 100% and (d) 1% of the volume of the spinal cord. In Fig. 5(c), an EQD_2_ of 52 Gy gives an NTCP of 5% for T_1/2_ = 0, but this value decreases for T_1/2_ over 3 h and reaches at least 44 Gy (T_1/2_ = 10 h). In Fig. 5(d), an EQD_2_ of 58 Gy gives an NTCP of 5% for T_1/2_ = 0, but this value decreases for T_1/2_ over 3 h and reaches at least 49 Gy (T_1/2_ = 10 h).

## Discussion

In this study, the impact of incorporating the IR model on NTCP was evaluated by retrospectively planning CSI for six patients. It should be noted that the study needs more cases to confirm the results because of a limited number of samples. Table 2 summarizes the results in this study.

**Table 2:** Characteristics between small T_1/2_ vs large T_1/2_

	small T1/2		large T1/2
Cell repair assumption	fast	↔	slow
NTCP	low	↔	high
Treatment evaluation	underestimate	↔	overestimate (safer)
Schedule of junction shift	independent	↔	dependent

For CSI planning, NTCP with T_1/2_ > 6 h was significantly higher than NTCP with T_1/2_ = 5 h, and it increased as T_1/2_ increased, particularly with a gap size of − 5 mm. Therefore, the setup of patients for CSI should be conducted carefully, particularly for patients having variations in the number of vertebrae. The increase of T_1/2_ with the IR model provides a long time for cells to be repaired from radiation damage, thus resulting in high NTCP. Under existing circumstances wherein wide 95% CI of T_1/2_ are reported, the largest conceivable value of T_1/2_ should be applied to evaluate the NTCP on the safest side because it largely depends on the value of T_1/2_. In the future, we need to search for an exact value of T_1/2_ not for rats but for humans to confirm the NTCP of cell repair. T_1/2_ for a carbon-ion beam was reported to be shorter than that for a photon beam [17]. Hence, T_1/2_ should also be investigated for different types of particle radiation beams.

Additionally, we examined the dependence of the NTCP on the junction-shift schedule (Fig. 4). For gap sizes of 5 and 0 mm, the NTCP did not vary with the junction-shift schedule because the maximum dose outside the junction regions was almost unchanged by junction shifts. However, for a gap size of − 5 mm, the NTCP decreased with changing schedules from reference plans to daily plans; we attribute this result to the hot spot in the junction area. For example, with the biweekly plan, an over-dose continued for two weeks and a subsequent mid-dose continued for a further two weeks on a certain point in the junction area, thus restraining cell repair. In contrast, with the daily plan, the over- and mid-doses were alternated every day on a certain point in the junction area, thus fostering cell repair and resulting in a significantly reduced NTCP. In particular, a larger T_1/2_ resulted in a larger reduction of NTCP because of greater cell repair. A deviation in NTCP associated with different schedules was seen because of tissue with comparatively long T_1/2_, similar to the case for the spinal cord.

According to the results of manually calculating the NTCP considering the IR model, as shown in Fig. 5(a)-(b), the increase in NTCP due to the increase of T_1/2_ became prominent for an EQD_2_ of 60–70 Gy, which corresponds to a dose with a steep normalized gradient of the dose response curve. By contrast, the NTCP hardly increased as T_1/2_ increased for an EQD_2_ of 30–40 Gy as irradiated in CSI planning; this result is consistent with the results of CSI planning demonstrated in this study.

The EQD_2_ with an NTCP of 5% was also calculated with IR model for T_1/2_ ranging from 1 h to 10 h (Fig. 5(c)-(d)) which can be considered the tolerance dose for normal tissue. Assuming whole-organ irradiation (the entire spinal cord), the tolerance dose of 52 Gy (T_1/2_ = 0 h) was decreased by 15%, reaching at least 44 Gy (T_1/2_ = 10 h). Assuming partial organ irradiation (1% volume), the tolerance dose of 58 Gy (T_1/2_ = 0 h) was decreased by 15%, reaching at least 49 Gy (T_1/2_ = 10 h). To think on the worst case supposed in this study, the dose should be under 44 Gy for whole-organ irradiation and under 49 Gy for partial (1%) organ irradiation.

Although the NTCP can estimate complications with normal tissue, the parameters of the LQ model and NTCP used in this study contain uncertainties. For example, the α/β value of 0.87 is reported in several papers [18, 19], which is smaller than the value used in this study. In this case, the NTCP could depend on T_1/2_ more largely, and increase more notably as T1/2 increased, because the value of β is related to the repairable damage as shown in eq. (6). Therefore, the α/β value has an impact on NTCP. Further studies are needed in order to estimate the dependency on the α/β value. Furthermore, other NTCP models have been proposed, such as the Lyman–Kutcher–Burman model [20, 21], and the absolute value of the NTCP depends on those models. However, the relative effects on the NTCP of incorporating the IR model should have the same tendency irrespective of parametric uncertainty or NTCP model.

Although we used a mono-exponential repair model that assumes a single repair constant (i.e., the repair half-time), other studies have reported a bi-exponential repair model that assumes two repair kinetics with long and short repair components [5, 6, 22, 23]. However, Levin-Plotnik et al. detected no difference between the mono- and bi-exponential repair models because the time to complete repair is affected only by the long repair component after an inter-fractional time of 5 h when the short repair component is saturated [24]. Therefore, our results with the mono-exponential model should not be markedly different from those with the bi-exponential repair model for actually used inter-fractional times, such as 24 h. Even if the bi-exponential model were to fit the experimental data, determining the length of the long repair component is more important than that of the short one because the proportion of damage repaired by the long component is larger than that repaired by the short component, as suggested by Ang et al. [5].

In contrast to physical dosimetric criteria, biological criteria, such as the NTCP, are advantageous to be able to account for not only the cell repair effect but also for several radiobiological phenomena such as clonogenic cell density, radiosensitivity, and hypoxia [25]. By incorporating these biological phenomena into the figures of merit, biologically based treatment planning could increasingly reproduce treatment outcomes toward biologically accurate radiotherapy.

## Conclusions

Incorporating an IR model allows the NTCP to be evaluated including the effect of cell repair and to reflect the effect of scheduling. However, the NTCP is significantly affected by the repair half-time T_1/2_. In the future, using precise values of T_1/2_ for humans according to the radiation used, biologically based treatment planning using NTCP values that include the of cell repair effect may be clinically performed.

## Abbreviations

CI: confidential interval
CSI: craniospinal irradiation
EQD2: equivalent dose in 2 Gy fractions
IR: incomplete repair
LQ: linear-quadratic
MLC: multi-leaf collimator
NTCP: normal tissue complication probability
